# Optimizing Production of Antigens and Fabs in the Context of Generating Recombinant Antibodies to Human Proteins

**DOI:** 10.1371/journal.pone.0139695

**Published:** 2015-10-05

**Authors:** Nan Zhong, Peter Loppnau, Alma Seitova, Mani Ravichandran, Maria Fenner, Harshika Jain, Anandi Bhattacharya, Ashley Hutchinson, Marcin Paduch, Vincent Lu, Michal Olszewski, Anthony A. Kossiakoff, Evan Dowdell, Akiko Koide, Shohei Koide, Haiming Huang, Vincent Nadeem, Sachdev S. Sidhu, Jack F. Greenblatt, Edyta Marcon, Cheryl H. Arrowsmith, Aled M. Edwards, Susanne Gräslund

**Affiliations:** 1 Structural Genomics Consortium, University of Toronto, MaRS South tower, 101 College street, Toronto, ON M5G 1L7, Canada; 2 Department of Biochemistry and Molecular Biology, Knapp Center for Biomedical Discovery, University of Chicago, 900 East 57th St., Chicago, IL 60637, United States of America; 3 Terrence Donnelly Center for Cellular & Biomolecular Research, University of Toronto, 160 College Street, Toronto, ON M5S 3E1, Canada; 4 Department of Molecular Genetics, University of Toronto, 1 Kings College Circle, MSB-4180, Toronto, ON M5S 1A8, Canada; Vrije Universiteit Brussel, BELGIUM

## Abstract

We developed and optimized a high-throughput project workflow to generate renewable recombinant antibodies to human proteins involved in epigenetic signalling. Three different strategies to produce phage display compatible protein antigens in bacterial systems were compared, and we found that *in vivo* biotinylation through the use of an Avi tag was the most productive method. Phage display selections were performed on 265 *in vivo* biotinylated antigen domains. High-affinity Fabs (<20nM) were obtained for 196. We constructed and optimized a new expression vector to produce *in vivo* biotinylated Fabs in *E*. *coli*. This increased average yields up to 10-fold, with an average yield of 4 mg/L. For 118 antigens, we identified Fabs that could immunoprecipitate their full-length endogenous targets from mammalian cell lysates. One Fab for each antigen was converted to a recombinant IgG and produced in mammalian cells, with an average yield of 15 mg/L. In summary, we have optimized each step of the pipeline to produce recombinant antibodies, significantly increasing both efficiency and yield, and also showed that these Fabs and IgGs can be generally useful for chromatin immunoprecipitation (ChIP) protocols.

## Introduction

Antibodies are widely used in the scientific community, as therapeutic agents and research tools. There are more than 500,000 commercially available antibodies on the market today, but many of those target a small number of popular proteins [1]. Most are not well described and only about a third actually recognize their targets specifically [2–4]. This leads to enormous problems in reproducing biomedical research. It is generally agreed that the availability of well-defined high quality antibodies for each human protein would greatly increase the quality and reproducibility of biomedical research [5].

The Human Protein Atlas is progressing toward the goal of covering the complete human proteome with high quality antibodies, having already generated selective polyclonal antibodies for >15,000 human proteins, and having tested their efficacy in a range of assays [6, 7]. It is widely agreed that these reagents will need to be complemented by well-characterized and sequence-verified renewable reagents, such as monoclonal antibodies or recombinant antibodies of various scaffolds. To this end, projects to generate scFvs recognizing surface receptors [8], recombinant antibodies to transcription factors [9], recombinant scFvs and Fabs for SH2 domains have been reported [10] and monoclonals for selected human proteins have been described or are currently underway (https://commonfund.nih.gov/proteincapture/index).

Progress at pilot scales has been encouraging, and there is little doubt that given a well-folded antigen, it is possible to generate a recombinant affinity reagent with desired properties to be suitable for a given assay or experiment. Arguably, the hurdles to large-scale generation of recombinant affinity reagents are thus more about logistics and efficiency than about feasibility. For example, many projects employed antigen proteins already produced for other purposes—can new antigens be produced cost-effectively and at appropriate throughput? The expression of recombinant Fabs in *E*. *coli* is known to be rather low, which makes scale up and purification difficult and costly—can recombinant Fab expression be improved in order to allow for automated purification? The characterization of each monoclonal, scFv or Fab in cell biology assays is time consuming, expensive and often bespoke—can cell-based validation be streamlined?

To address these questions and generate tool reagents to study the regulation of chromatin remodeling, we embarked on a project to generate renewable Fabs for the enzymes and protein interaction modules that are involved in regulating epigenetic signalling. Key players in this process are the enzymes that contain domains that read (bromo-, chromo- and tudor domain containing proteins), write (acetylases, methyltransferases, ubiquitinases) or erase (deacetylases, demethylases, deubiquitinases) histone post-translational modifications. For this project, 265 domains from such proteins were targeted as antigens to generate highly selective and well-characterized affinity reagents (Table 1). Fabs against these target domains were generated by phage display mutagenesis selections employing a high performance reduced genetic code library initially developed by Sidhu and coworkers [11] and further refined and used in these studies [12, 13]. The selected Fabs were first tested *in vitro* by competitive phage ELISA, followed by cell-based assays on the most promising candidates either as biotinylated Fabs or as hybrid IgG molecules with the human Fab scaffold fused to the Fc part of mouse IgG1. Here, we describe an improved process that increased yield, purity and quality of antigen, and the Fab and IgG reagents. We also demonstrate the versatility of the resulting Fab antibodies in uses for various cell-based assays on endogenous full-length protein.

**Table 1:** Overview of targets and their success rates.

*Protein Family Name*	*Domains produced as antigens*	*Domains with Fabs selected and tested*	*Domains with validated antibodies*	*Protein Family success rate*
Acetyltransferases	7	3	0	-
Bromodomains	40	35	23	66%
Chromatin assembly and remodeling	7	2	2	-
Chromodomains	27	26	16	62%
CXXC domains	3	1	0	-
Deacetylases	7	5	4	-
Demethylases	13	12	10	83%
MBT domains	11	11	11	100%
Misc.	4	3	3	-
Methyltransferases	48	36	19	53%
PARPs	13	8	3	38%
PHD domains	16	5	3	-
PWWP domains	9	7	6	86%
SANT domain containing	5	3	3	-
Small RNA Pathways	2	1	1	-
Tudor domains	27	20	7	35%
Ubiquitin-related	15	3	2	-
WD40 repeat proteins	11	5	5	100%
**Total**:	**265**	**186**	**118**	**63%**

Overview of the 265 antigen domains included in the study and what protein families they belong to. Family success rate was calculated as percentage of targets that were successful in selections and which also yielded at least one antibody that passed cell-based validation.

## Results

### Antigen production

**Fig 1:**
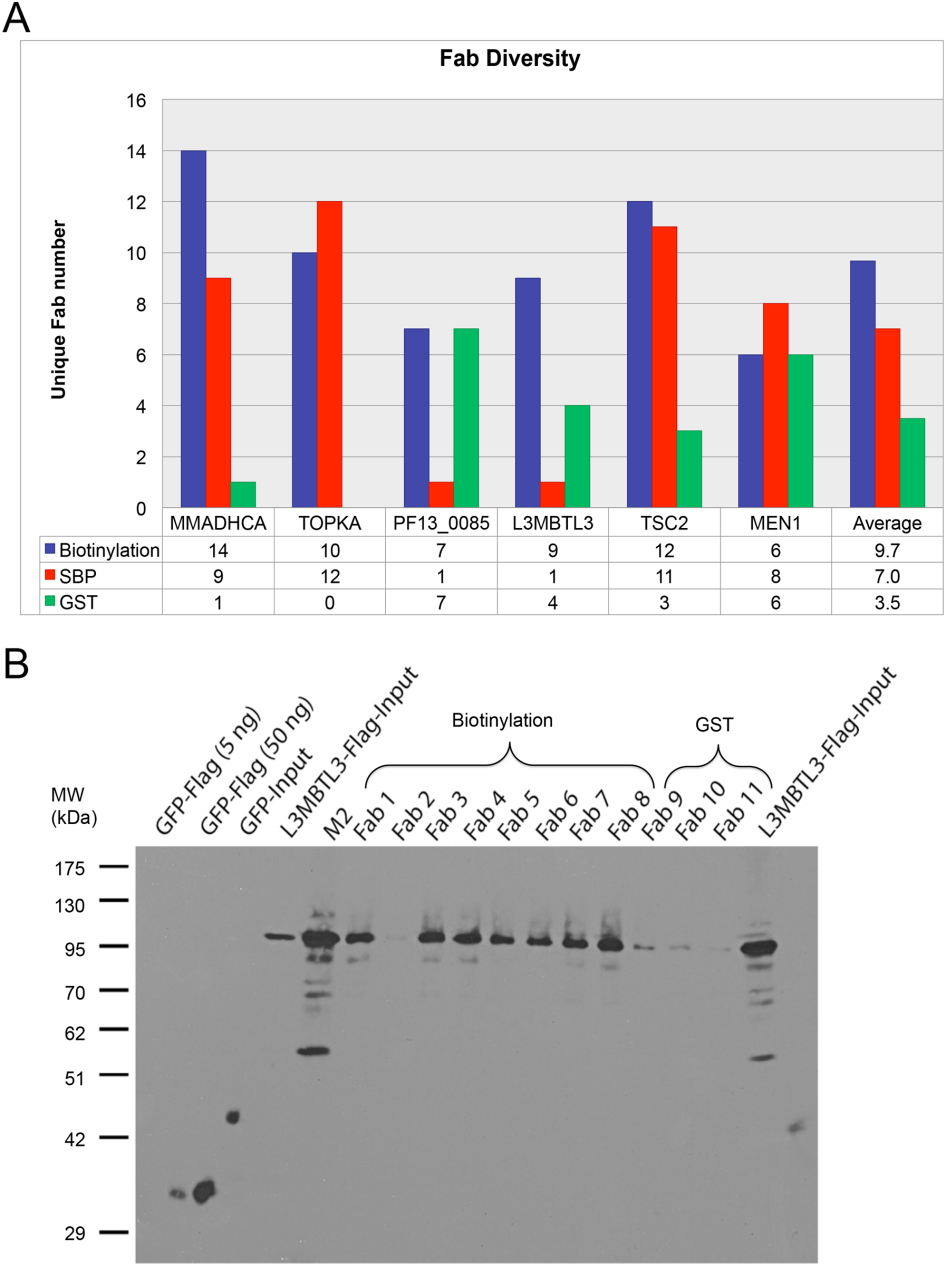
Comparison of antigen immobilization methods. (A) Three different affinity tags were tested for antigen immobilization in phage display; *in vivo* biotinylation through an Avi tag, SBP and GST tags. The diversity of Fabs derived from these differentially tagged antigens was then compared. (B) Immunoprecipitation with Fabs selected against either Avi-tagged antigen or GST-tagged antigen from a cell lysate expressing FLAG-tagged target protein. Immunoprecipitated antigen was detected with an M2 antibody against the FLAG tag. Fabs selected against Avi-tagged antigen generally show a higher recovery of the antigen.

The selection of recombinant antibodies requires high-quality, stable and well-folded antigens. For *in vitro* selection, the antigen also needs to be appended with a tag to enable immobilization on a surface during selections. We set out to determine which expression system would best generate antigens with the desired properties. We first compared the efficiencies of Fab selection using six different antigens appended with three different tags each (Avi tag, Glutathione S-transferase (GST) or Streptavidin-binding peptide (SBP). This analysis showed that an *in vivo* biotinylated Avi tag was the most successful option; we were able to select a pool of Fabs for all six targets appended with an Avi-tag, whereas we got more than a single Fab for only four antigens appended with either GST or SBP (Fig 1A). Also, the Fabs selected using Avi-tagged antigens performed generally better in validation (Fig 1B).

After selecting the Avi-tag strategy, 350 constructs were designed based on information from an internal database of well-expressing domains [14, 15] and then subcloned into an *E*. *coli* expression vector (p28BIOH-LIC), which appended a C-terminal hexahistidine tag and an N-terminal Avi tag enabling *in vivo* biotinylation. We also explored using a vector (pNIC-Bio2) appending a decahistidine tag because, in theory, this tag would offer higher affinity immobilization to an NTA chip in surface plasmon resonance (SPR), which could facilitate acquiring more accurate kinetic binding data for the Fabs. However, the yields of soluble antigen protein were about 5-fold lower in pNIC-Bio2 compared with p28BIOH-LIC (data not shown). Thus, despite having a lower-affinity His tag, the use of p28BIOH-LIC greatly increased the cost-effectiveness and efficiency of the process.

Of the ~350 domains cloned from about 300 target proteins, 265 could be purified to >90% purity and mono-dispersity. (Table 1). The other 85 proteins did not come off the size exclusion chromatography (SEC) column in symmetrical peaks indicating they did not represent monodisperse proteins and thus were not pure enough or gave extremely low yields. The identities of the proteins were verified and the efficiency of biotinylation assessed using mass spectrometry. All 265 antigens that could successfully be produced were also more or less completely biotinylated. The biotinylated antigens were then used to select recombinant Fab fragments using phage display library selection protocols that have been described [11–13, 16, 17]. The antigens used in these selections were often from frozen stocks. To ensure that the antigen was stable to freeze/thaw conditions, thawed antigens were retested in analytical SEC to ensure they remained monodisperse. Approximately 10% of the antigens were found to be incompatible to freeze/thaw conditions.

### Phage display selections and primary validation

Prior to starting phage display selections, antigens were checked for aggregation to ensure high quality for both the phage display sorting process and the initial Fab validation step. Initial Fab clones were picked based on either phage ELISA [11] or single point competitive phage ELISA assay calibrated to select clones with best specificity and affinity (S1 Fig) [16]. While some binders were tested in SPR generating kinetic and affinity data, most were subjected only to single point competitive ELISA assay. This method has proven to be a good predictor of specificity and affinity especially when implemented high-throughput format (S1 Fig). Only Fabs with desired properties and a predicted high affinity for the purified antigen (K_D_ < 20 nM) were carried forward for further validation. Where possible, the high affinity Fabs were counter-screened for cross-reactivity to the close structural homologs (family members) of the target antigen and when found these cross-reacting antibodies were eliminated from the pool.

Over 900 Fabs for 196 antigens (~4.5 Fabs per antigen) were successfully selected and passed these initial screens, and were advanced for further validation in cell-based assays. Most selections provided 1–20 binders for each antigen. However, we chose to examine only the five best Fab candidates per antigen, based on the ELISA results, in cell-based assays because the cell-based validation pipeline had a more limited capacity.

### Fab production

**Fig 2:**
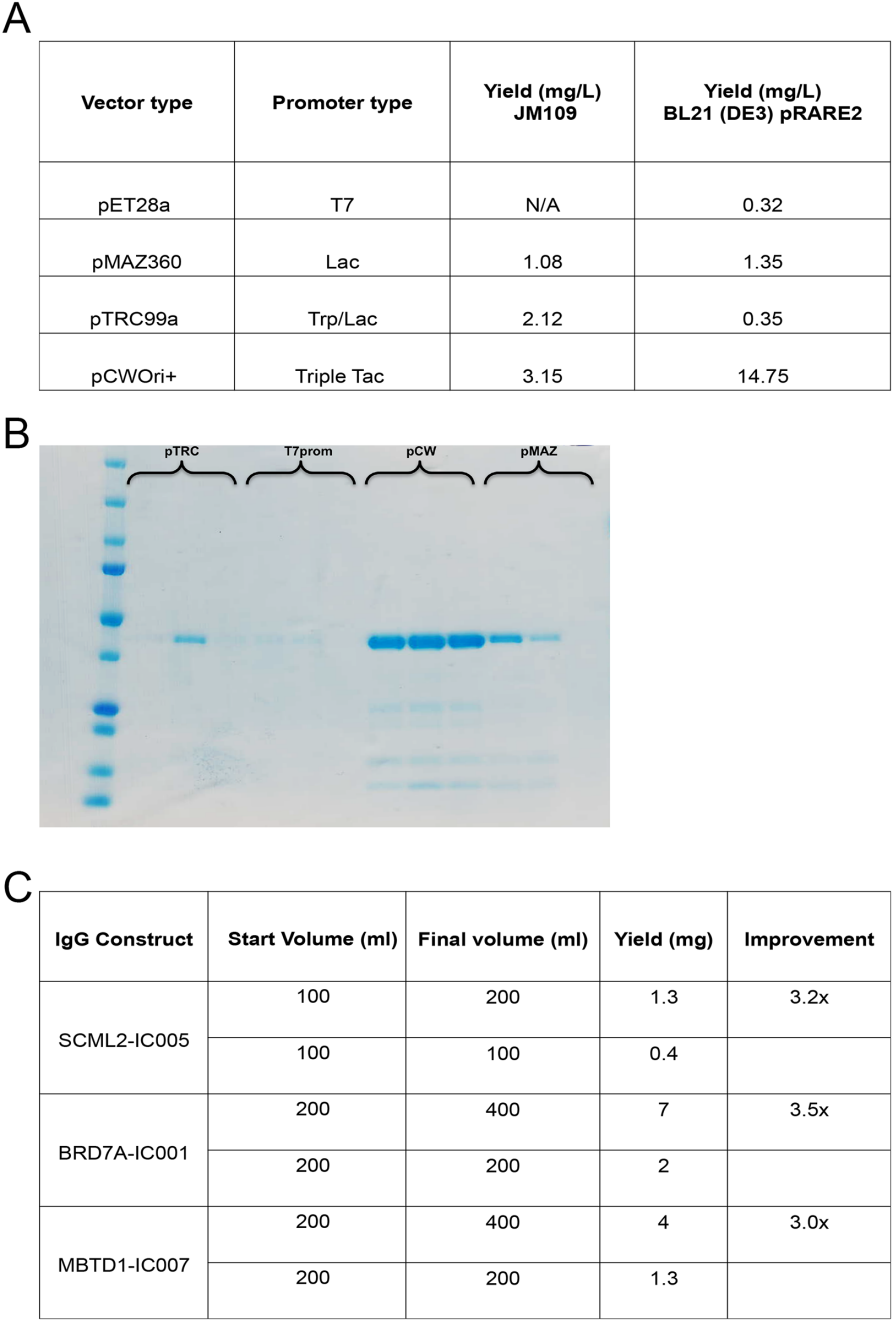
Fab and IgG production. (A) Comparison of purification yields between different expression vectors using an anti-MBP Fab as an example. The large-scale purification method on the ÄKTA Xpress including a heat denaturation step was used. (B) SDS-PAGE gel showing the anti-MBP Fab produced with various expression vectors and purified in triplicate. (C) IgG production yields with and without the dilution strategy.

Fabs are readily produced in *E*. *coli*, but levels of Fab expression in published phoA-driven vectors [18] had low and variable yields. Thus, we set out to identify vectors that would increase expression levels and simplify handling in a higher-throughput setting. Four different promoters were compared: pT7 from pET28a [19], pLac from pMAZ360 [20], pTrc from pTRC99a (a hybrid of the *trp* and *lac* promoter systems) [21] and a triple version of the *tac* system from the expression vector pCWOri+ [22–24]. The pCWOri+ vector has been used extensively to produce cytochrome proteins in bacterial hosts [24]. We also compared two different *E*.*coli* strains: BL21 (DE3) pRARE2, which provides tRNAs for codons rarely used in *E*.*coli* (Novagen, Merck Biosciences); and JM109, which is often used for production of secreted proteins. The optimal combination (pCWOri+ with BL21 (DE3) pRARE2) significantly increased the yield of a previously selected anti-MBP Fab using single step purification with protein A Sepharose beads (Fig 2A and 2B).

Based on our evaluation, we constructed a new expression vector, pSFV4 (Genbank KC164372), which was designed to contain as much of the Fab scaffold as possible. By subcloning only the unique sequences of the selected Fab heavy and light chains cloning errors could be minimized. The default version of pSFV4 adds an Avi tag for *in vivo* biotinylation on the heavy chain C-terminus. We observed that smaller fragments of Fabs, single heavy and light chains for example, are often produced and co-purified on protein A-sepharose. Because our recombinant Fab scaffold is extremely stable (Tm ~ 80°C), and Fab degradation fragments have a lower melting point than the intact Fab and precipitate upon heat treatment [16], we introduced a heat denaturation step that effectively removes the degradation fragments during purification (S2 Fig). Biotinylated Fabs were produced both in small-scale for validation (average yield 80 μg/8 ml or 10 mg/L) and in large-scale for distribution (average yield 4 mg/L). A more thorough purification method was used for large-scale production, which resulted in final yields that were lower than in the small-scale productions, but still a significant improvement when compared to previous expression vectors and protocols.

### IgG production

We explored the ease of converting the recombinant Fabs to recombinant IgG formats as these might be preferable over Fabs in some cases. To construct recombinant IgGs, sequences from the recombinant Fabs were transferred to two expression vectors, one for the light chain and one for the heavy chain where the latter was fused to an Fc portion from mouse IgG1. IgGs were produced both in small-scale (10–40 mL) and in large-scale (200–400 ml) by transient transfection of suspension-grown HEK293F cells. Transfection was performed in high-density cultures that were diluted twice after 24 hours; this resulted in a 3-fold improvement in IgG yield (Fig 2C). Initially polyethylenimine (PEI) was used as transfection reagent in order to improve cost-effectiveness, but this protocol resulted in significantly lower yields than with 293Fectin™ (Life Technologies), and ultimately was not the most cost-effective approach (data not shown). In small-scale, IgGs were purified from the cultivation medium using a batch binding strategy with protein A Sepharose beads (LTC) and with an average yield of 15 mg/L. In large-scale, IgGs were purified using either the same batch binding strategy or HiTrap Protein A columns on an ÄKTA Xpress system (GE Healthcare). Yields were almost doubled, from 15 mg/L to 25 mg/L, using the ÄKTA Xpress strategy. Theoretically, mouse IgG1 should have a higher affinity for protein G than for protein A (https://www.millipore.com/techpublications/tech2/binding_properties), but in a direct comparison using protein A and protein G Sepharose beads (LTC), the yields were higher from protein A beads (data not shown).

### Characterization in cell-based assays

Ultimately, the usefulness of a pipeline and the quality of reagents it generates, can only be evaluated by assessing a measurable output in a relevant application. We thus tested the efficiency of each Fab to immunoprecipitate its native antigen in the context of cell lysates. Initial experiments were performed on HEK293 cell lysates expressing recombinant, FLAG-tagged versions of some of the antigens. After immunoprecipitation with the recombinant Fabs, the presence of the FLAG-tagged antigen was detected by an antibody against the FLAG tag. We also developed a semi-quantitative mass spectrometry approach to assess if the endogenously expressed target antigen was immunoprecipitated by the Fab [25]. This procedure also allowed us to assess the levels of all co-immunoprecipitating proteins.

**Fig 3:**
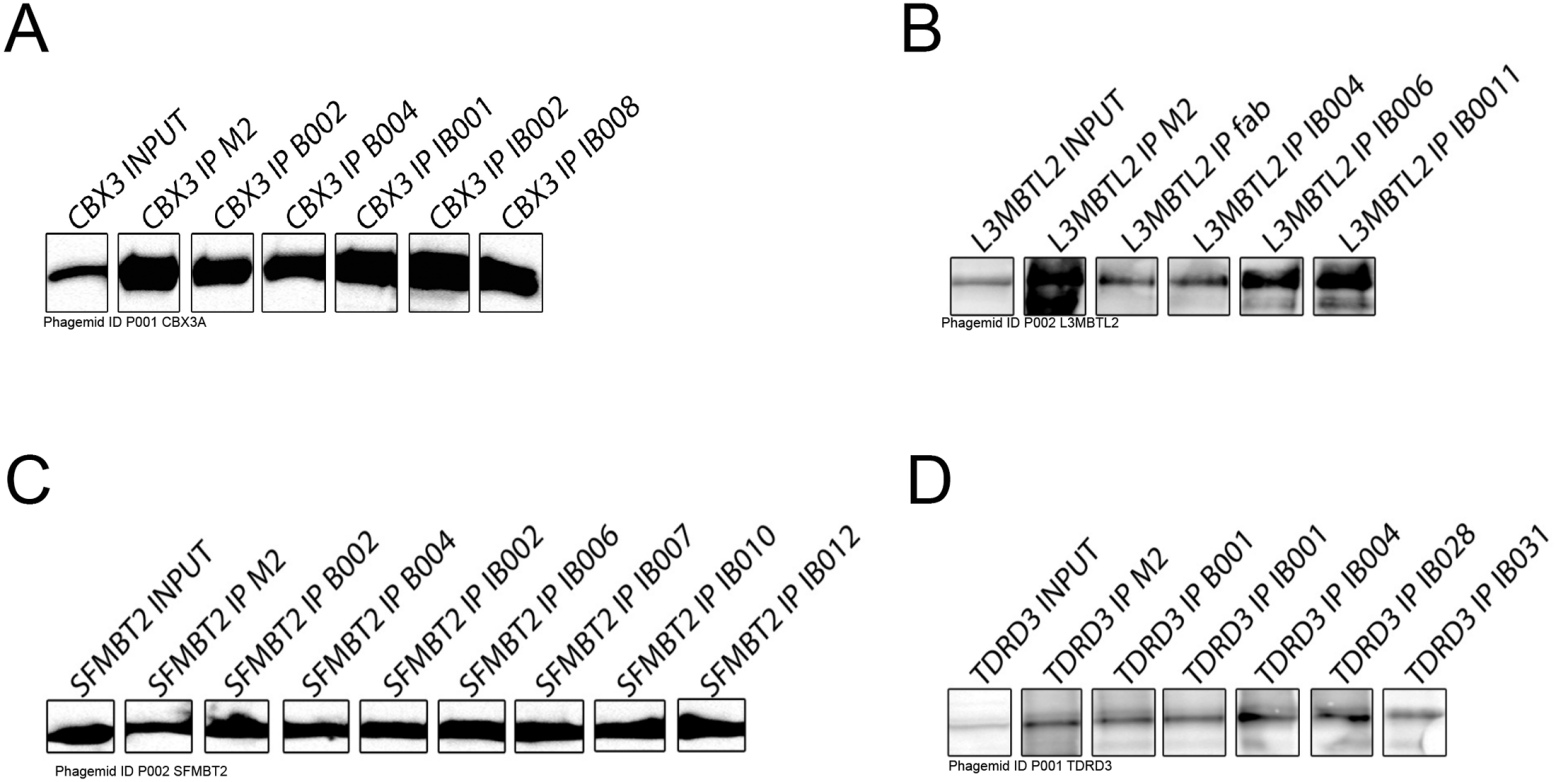
Performance consistency among Fabs and IgGs generated against the same target. Multiple Fabs and IgGs against several targets were used to immunoprecipitate their corresponding FLAG-tagged antigens. Western blot was performed and the presence of the FLAG-tagged immunoprecipitated protein was detected with an antibody against the tag. A) CBX3. B) L3MBTl2, C) SFMBT2, D) TDRD3. FLAG-tagged GFP was used as control (data not shown). Fab batches are labeled with a trailer “-Bxxx” and IgG batches are labeled with a trailer “-IBxxx”. Fabs against CBX3 and SFMBT2 have been produced twice (CBX3 (B002, B004); SFMBT2 (B002, B004)) while Fabs against L3MBTL2 and TDRD3 have been produced only once (L3MBTL2 (B001); TDRD3 (B001)). Multiple IgGs have been produced with corresponding IB numbers. Fabs/IgGs derived from the same phagemid clone have similar efficiencies and show a high lot-to-lot consistency.

We analyzed 811 Fabs (for 186 antigens) by one or both of these methods. 407 unambiguously immunoprecipitated their cognate antigens from cell lysates. These 407 Fabs corresponded to 118 antigens, or 63% of the antigens tested (Table 1). We also observed that Fabs and IgGs produced from the same phagemid exhibited similar immunoprecipitation efficiencies and batch-to-batch variability was negligible (Fig 3). These results suggest that recombinant Fab antibodies are effective in recognizing their cognate antigens in cell lysates. Many of these reagents also proved suitable for immunofluorescence (IF) [25].

Chromatin immunoprecipitation (ChIP) is another application where data quality is largely dependent on the antibody quality and where there is great need for well-characterized, specific and selective antibodies. ChIP is an immunoprecipitation related method, but it involves crosslinking of protein to DNA and the experimental conditions differ from those used in standard immunoprecipitation experiments. There are many technical challenges in performing ChIP assays and the required level of analysis goes beyond the scope of this study. However, to get a preliminary assessment of the suitability of the Fabs as ChIP reagents, we determined if the Fabs were able to immunoprecipitate full FLAG-tagged antigen under ChIP-like immunoprecipitation conditions (S3A Fig). Instead of digesting proteins and isolating DNA, we simply took the immunoprecipitated material, performed western blotting and tested for the presence of the FLAG-tagged full-length antigen using anti-FLAG antibody (S3B Fig). We used ChIP-qPCR for couple of targets with known genomic loci and showed that these targets are in fact enriched at these sites (S3C Fig). We found that most of the antibodies that immunoprecipitate their endogenous antigens from cell lysates were also compatible with ChIP protocols.

## Discussion

**Fig 4:**
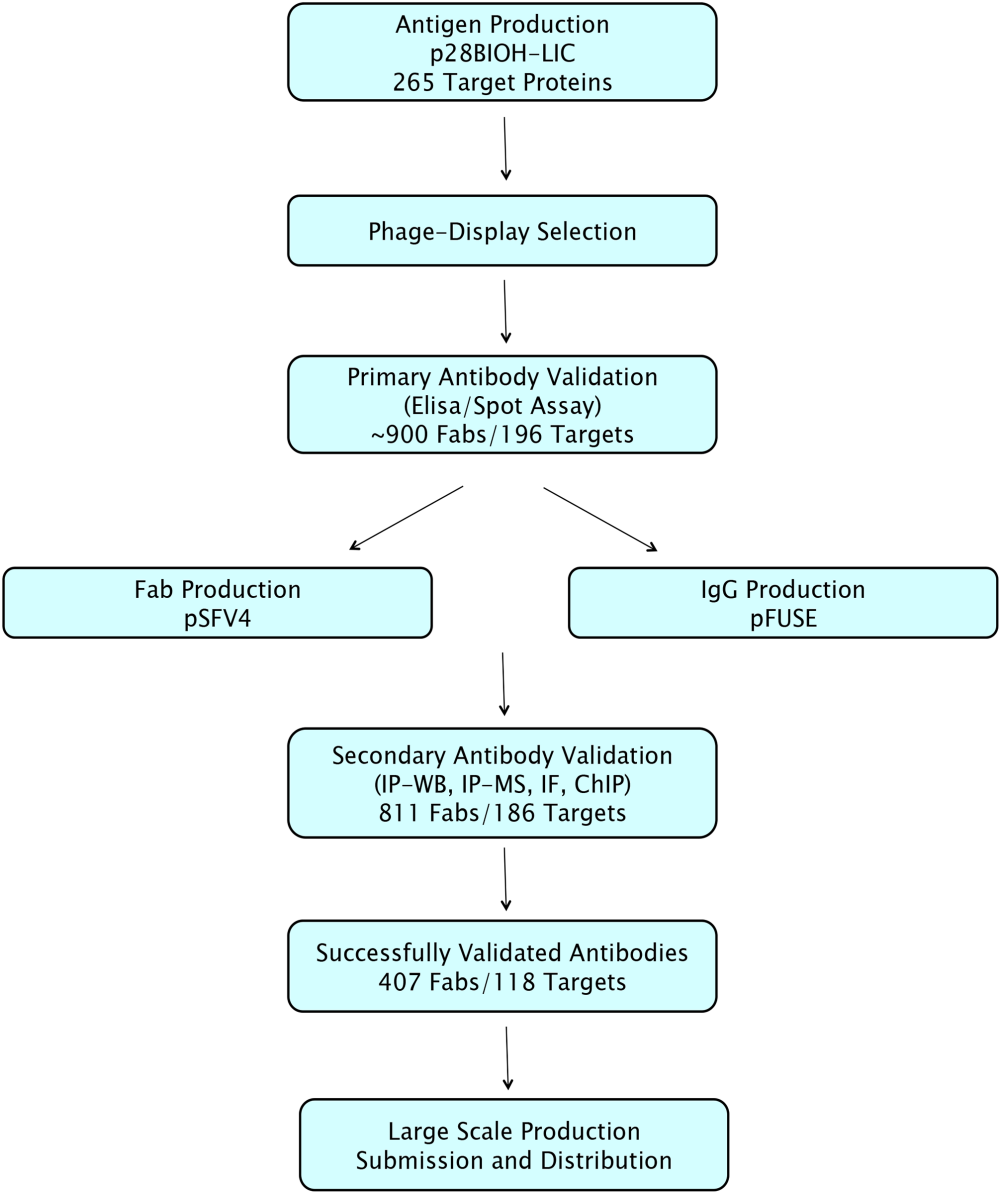
Pipeline overview. Overview of the project pipeline from antigen production to cell-based validation, either on Fabs or IgGs.

We established and optimized a pipeline to generate renewable and reproducible recombinant antibodies against protein targets that can be expressed in soluble and well-folded form. The overview of the pipeline is presented in Fig 4. Compared with published protocols, the optimized process increased the number of antigens produced, the yields of antigen, Fab, and IgG materials. The process also generated Fabs and IgGs shown to work in cell biology applications, such as immunoprecipitation and chromatin immunoprecipitation (ChIP-qPCR). Since this was a pilot study, many people have been involved in optimizing the various steps in the pipeline. For running it continuously however, 4 FTEs working with antigen/antibody cloning, antigen/antibody production, phage display selections and cell-based validation respectively, would be able to process around 20 antigens per month, from antigen cloning to a validated Fab. The overall lead time of these steps would be approximately 4 months. Conversion to IgG format, IgG production and re-validation would add approximately another 3 months.

Access to good antigens is a major bottleneck in the making of recombinant antibodies. For this project, we exploited the extensive internal database at the SGC that contains test expression data for a large variety of targets. This information guided the design of most of the constructs, but some of the antigens used in this study represented completely novel targets. Even though all the constructs had to be re-cloned and produced with a new tag set-up, we were able to efficiently produce a sufficient number of high-quality antigens.

Another aim was to establish robust protocols suitable for high-throughput antibody production. After investigating various systems, we found that the promoter from pCWOri+ gave the highest yields, and used this information to design a new Fab expression vector, pSFV4. pSFV4 was tailor-made to optimize the transfer of the Fab sequences from the phagemid, in the originating library independent fashion, to the expression plasmid with a minimal amount of changes. The pSFV4 vector also allowed the tag to be changed with a simple restriction/re-ligation procedure. For antibody characterization, we used biotinylated Fabs to enable simple and streamlined detection by streptavidin-conjugates, but we also made His-tagged and untagged Fabs for co-crystallization purposes.

In this study, we produced antigens in *E*.*coli*, but we also produced *in vivo* biotinylated antigens in the baculovirus expression system using Sf9 insect cells. These antigens performed well in phage display selections, although the Fabs have not yet been subjected to cell-based validation. IgG production using transient transfection of mammalian cells has worked reasonably well in this project, but there are some notable drawbacks. The cost associated with mammalian production is higher than for Fabs produced in *E*. *coli*, yields can be very low for some IgGs and some IgGs are prone to precipitate after purification. We have started to explore the newly launched Expi293F system (Thermo Fisher) for production of recombinant IgGs; this system shows improved yields and culture viability. Generally, Fabs and IgGs derived from the same phagemid show very similar behaviours. In most cases the IgGs give a stronger signal than the Fabs, which might be expected, but Fabs usually give a strong enough signal to be used in practice. It is up to the user if the small boost in performance given by moving to the IgG format is warranted. Our conclusion is Fabs can be used for most applications, and the time-consuming and costly procedure to convert the Fabs to IgG can be avoided.

Fabs generated to recombinant antigen domains purified from bacteria bind well to the domains they were raised against, but only two-thirds could precipitate their full-length antigen from HEK293 cell lysates. The one third of the Fabs that did not perform well either recognized an epitope that is masked in the full-length protein or targeted an antigen that was not expressed in HEK293 cells. This finding underlies the importance of antibody validation within the natural environment of its target protein.

We generated antibodies against members of several diverse protein families. For reasons we do not yet understand, some protein families generated more IP-competent recombinant Fabs than others (Table 1). Other protein families, PRDMs for example, have many members that are expressed at very low levels or in specific cell types, so they are difficult to validate with our standard protocols.

## Materials and Methods

### Subcloning of antigens

Expression constructs for the intracellular production of antigens in *E*. *coli* with hexahistidine purification tags [26] and Avi tags for in vivo biotinylation [27] [28] were constructed in p28BIOH-LIC4 (GenBank KC164371). Antigen coding DNA sequences were subcloned from cDNA sources by PCR amplification using specific primers with the forward primer addition of (5’-gctggaggttcaggt–-3’) and reverse primer addition of (5’-atgaccacttccacc–-3’). PCR products were inserted into BseRI linearized vector using the In-Fusion Cloning Kit (Clontech) and verified by DNA sequencing. Antigens were also cloned into the vector pNIC-Bio2 (GenBank JF91291) as previously described [29].

### Overexpression and purification of antigens

The p28BIOH-LIC plasmids were transformed into *E*. *coli* BL21 (DE3) carrying a plasmid for co-expression of BirA ligase. The cells were cultured at 37°C in the LEX system (Harbinger Biotech, Markham, Ontario) in Terrific broth medium supplemented with 35 μg/L chloramphenicol, 50 μg/L Kanamycin and 50 μg/L D-Biotin until the OD_600_ of the cultures reached 3. The cultivations were cooled to 18°C and then protein expression was induced by adding isopropyl-β-D-thiogalactopyranoside to a final concentration of 0.5 mM. Incubation was continued at 18°C overnight, cells were harvested by centrifugation and stored at -80°C.

Cell pellets from 2 L culture were resuspended in 200 mL lysis buffer (50 mM Tris-HCl pH 8.0, 300 mM NaCl and 10 mM imidazole) and sonicated on ice. The lysate was clarified by centrifugation at 16,000 rpm for 60 min at 4°C. The soluble fraction was decanted and filtered through 0.45 μm filters and then loaded onto a 5 mL HiTrap Ni-chelating column (GE Healthcare) on an ÄKTA Xpress (GE Healthcare) equilibrated with the lysis buffer. After washing in 50 mM Tris-HCl pH 8.0, 300 mM NaCl and 10 mM imidazole to remove unbound fractions, the protein was eluted in 50 mM Tris-HCl pH 8.0, 300 mM NaCl and 250 mM imidazole. The eluate was then applied to a Hiload XK16/60 Superdex 200 column (GE Healthcare) equilibrated with 20 mM HEPES, pH 7.4, 150 mM NaCl, 0.5 mM EDTA and 0.5 mM TCEP. Relevant fractions corresponding to a mono-disperse peak were pooled and analyzed by SDS-PAGE and mass spectrometry. Antigen batches were flash frozen in liquid nitrogen and stored at -80°C before shipping to the Fab selection labs.

### Phage display and panning

Prior to starting phage display selections and screening proteins were routinely checked for aggregation after thawing using size exclusion chromatography. Biotinylation levels and efficiency of magentic bead capture were tested by incubating 5 μg of protein with 50 μL of Streptavidin MagneSphere particles (Promega), washing once with 50 μL of a buffer 50 mM TRIS, 250 mM NaCl, 1 mM DTT (pH 8.0), and running beads resuspended in 1X SDS loading buffer on a SDS-PAGE gel. Monodisperse antigens with at least 80% bead capture efficiency were promoted to phage display panning. Antigens which were stored for longer periods of time were also tested using Agilent 2100 Bioanalyzer. Purity, sizing and absolute quantitation data were compared over time and problematic antigens were further tested in differential scanning fluorimetry (DSF). This allowed us to maintain constant quality of antigens used both in phage display and initial Fab validation. Up to four rounds selection were performed on Avi-tagged antigens according to previously published protocols [11–13, 16] and newly designed phage libraries [12, 13]. Concentrations of target proteins used were adjusted to 100 nM in the first round and 10 nM and 5 nM in the subsequent rounds to ensure proper stringency in the panning process. During the first round 2x10^12^ phage particles suspended in PBST-BSA (PBS, 0.05% (v/v) Tween 20, 0.5% (w/v) BSA) were used. Incubation of the target with a library was performed in room temperature for at least 1h and followed by extensive washing steps and finished with elution as described previously. Targets which have shown significant (more than 20-fold) enrichment after round 3 were nominated for phage ELISA screening.

### Phage ELISA

*E*.*coli* XL1-blue (Stratagene) colonies containing phagemids were grown in 96-well format in 400 μL of 2xYT broth containing carbenicillin 100 μg/mL and 10^10^ pfu/mL of M13-KO7 helper phage (New England Biolabs) overnight at 37°C. Supernatants containing Fab-phage were diluted 5 to 20-fold in PBST-BSA with or without soluble competitor (20 nM non-biotinylated target protein) in total volume of 50 μL. After 1 h incubation at room temperature, the mixtures were transferred to neutravidin-coated plates pre-loaded with 50 μL of 20 nM biotinylated target and incubated for 15 min. The plates were washed with PBST-BSA and incubated for 30 min with horse radish peroxidase/anti-M13 antibody conjugate (GE Healthcare) (1:5000 dilution in PBST-BSA). The plates were washed, developed with 3,3’,5,5’-Tetramethyl-benzidine/H_2_O_2_ peroxidase substrate (Thermo Scientific) and quenched with 1 M H_3_PO_4_. Absorbance at 450 nm was determined and for each clone, the competition ratio was calculated by dividing the signal in the presence of non-biotinylated target by the signal in the absence of competitor. Based on the phage ELISA results, the best performing clones were sequenced and passed on to sub-cloning.

### Surface plasmon resonance

Interaction analyses were performed using a BIACORE 3000 (GE Healthcare) at 20°C. Purified, hexa- or decahistidine tagged antigens were immobilized on an NTA sensor chip. Running buffer contained: 10 mM HEPES, 150 mM NaCl, pH 7.4, 0.05% (v/v) Tween 20. Antigens were captured by injecting 5 μL of 20–80 nM protein solution at a flow rate of 5 μL/min and protein concentration was adjusted based on predicted molecular weight to obtain response not higher then 100 RU. Up to three blank injections were performed to ensure stability of the surface before analyte injections were started. For kinetic assay, two-fold dilution series of Fab starting at 10 nM were injected over the NTA chip surface at a flow rate of 30 μL/min to minimize mass transport effects for 150 s. The resulting responses were measured for 300 s after the injection finished. Following each sample injection, the NTA chip surface was regenerated with 50 μL of 100 mM EDTA solution at a flow rate of 50 μL/min. All conditions were tested at 5 different Fab concentrations, and each concentration was tested in triplicate. For initial assessment of affinity single injection at 50 nM was used and tested in triplicate. Data processing and kinetic analysis were performed using in Scrubber 2 (BioLogic software). All sensorgrams were double referenced using blank channel and buffer injections. For the determination of kinetic rate constants, data sets were fit to a simple 1:1 interaction model using nonlinear regression analysis.

### Sub-cloning phagemid sequences to Fab and IgG vectors

Expression clones for the secretion of Fabs into the *E*. *coli* periplasm were constructed in pSFV4 (GenBank KC164372). Partial bi-cistronic Fab coding sequences containing the light chain and heavy chain variable regions were PCR amplified from each phagemid using the primers pSFV4-FwdClone (5’- cgcaacttattactgtcagc–3’) and pSFV4-RevClone (5’- agacggtgaccagggttcc–3’). Since inserts originating from two different libraries used [12] [13] share the same bi-cistronic Fab expression cassette they could be amplified using the same set of primers. To remove light chain FLAG tags the light chain and heavy chain coding sequences were amplified separately by pairing pSFV4-FwdClone with IP-LC- Rev (5’-gttaattaacactctcccctgttgaag–3’) and pSFV4-RevClone with IP-HC-Fwd (5’-gagagtgttaattaactcgaggctgagc–3’) and then recombined in the vector. The PCR product(s) were inserted into Sph1 linearized expression vector using the In-Fusion Cloning Kit (Clontech). Expression clones for the IgG production in mammalian cells were constructed. Light chain variable coding sequence was PCR amplified from each phagemid using the primers Light-Fwd-Afe1 (5’-cagtccgtgtccagcgctg–3’) and Rev-LV2 (5’-tttgatctccaccttggtac–3’) and inserted into the expression vector pFUSE-LIGHT (GenBank KC176267). Heavy chain variable coding sequence was PCR amplified from each phagemid using the primers Fwd-HV2 (5’-attcggaggttcagctggtggag–3’) and Rev-HV2 (5’-gagacggtgaccagggttc–3') and inserted into the expression vector pFUSE-HEAVY (GenBank KC176268). IgGs expressed from the pFUSE-LIGHT and pFUSE-HEAVY vectors are hybrids of a human light chain gamma 1 and a human heavy chain immunoglobulin gamma 1 constant 1, human hinge region, and a mouse immunoglobulin gamma 1 heavy chain constant regions 2 and 3. Fab coding sequence was also subcloned into the vectors pFUSE2ss-CLIg-mk and pFUSEss-CHIg-mG1 (InvivoGen) to produce IgGs. All expression constructs were verified by DNA sequencing.

### Overexpression and purification of Fabs

The pSFV4 plasmids were transformed into *E*. *coli* BL21 (DE3) BirA strains. The cells were cultured at 37°C in the LEX system in the presence of 35 μg/L chloramphenicol, 50 μg/L Kanamycin and 50 μg/L D-Biotin until the OD_600_ of the cultures reached 3 and then protein expression induced by adding isopropyl-β-D-thiogalactopyranoside to a final concentration of 0.5 mM. After incubation at 25°C overnight with shaking, cells were harvested by centrifugation and stored at -80°C.

Cell pellets from 2L culture were resuspended in 200 mL lysis buffer (50 mM Tris-HCl pH 8.0, 300 mM NaCl and 10 mM imidazole) and sonicated on ice. The lysate was clarified by centrifugation at 16,000 rpm for 60 min at 4°C. The soluble fraction was decanted and filtered through 0.45 μm filters and then loaded onto a protein A column on an ÄKTA Xpress equilibrated with the lysis buffer. After washing with PBS to remove unbound fractions, elution buffer (100 mM Acetic acid) was applied. The eluate was neutralized by adding 1/10 volume of 1.0 M Tris-HCl, pH 9. The peak fractions were pooled and analyzed by SDS-PAGE and mass spectrometry. Prior to storage at -80°C, the Fabs were dialyzed against dialysis buffer (1x PBS, pH 7.4, 0.09% sodium azide).

### Overexpression and purification of IgGs

Suspension-grown HEK293F cells with viability over 95% in FreeStyle medium (Invitrogen, #12338–018) were cultivated at 37°C under 8% CO_2_ atmosphere and shaking at 125 rpm on an orbital shaker incubator (VWR symphony Air-Jacketed CO2 Incubators, Models 5.3A and Thermo Scientific MaxQ 2000). Upon reaching a density of 1.5–1.8×10^6^ viable cells/mL, cells were transfected with a mixture of heavy chain and light chain plasmid DNA (total 2 μg/mL) at a 1:1 ratio using 293Fectin™ (Life Technologies) at a DNA:293Fectin™ ratio of 1:1. After 24 hours, the transfected cells were diluted twice with pre-warmed media containing Tryptone to a final concentration of 0.25%. Six to seven days after transfection, the culture supernatants containing the secreted proteins were harvested by centrifugation at 38,000 x *g* at 4°C, which removed cells and debris.

The pH of the supernatants was neutralized by the addition of 1/10 volume 10x PBS and filtered through 0.45 μm filters. The samples were then loaded onto 5 ml HiTrap Protein A columns on an ÄKTA Xpress equilibrated with lysis buffer. After washing with wash buffer (1x PBS, pH 7.4) to remove the unbound fractions, elution buffer (100 mM Acetic acid) was applied. Eluates were neutralized by the addition of 1/10 volume of 1.0 M Tris-HCl, pH 9 and the relevant fractions were pooled and analyzed by SDS-PAGE and Mass spectrometry. The verified IgGs were dialyzed against 500-fold volume of dialysis buffer (1xPBS, pH 7.4, 0.09% sodium azide). The final samples were diluted to a concentration of 0.5 mg/mL and then flash frozen by liquid nitrogen and stored at -80°C.

### IP-Western Blot

Generation of stable HEK293 cell lines overexpressing FLAG-tagged full-length antigens was previously described elsewhere [30]. Frozen cell pellets were thawed in High Salt AFC buffer (10 mM TRIS pH 7.9, 420 mM NaCl, 0.1% NP–40) and subjected to three cycles of freeze/thaw. Following sonication (5x, 0.3 s on/0.7 s off per 1 mL), the solution was treated with benzonase nuclease for 30 minutes and then clarified by centrifugation at 16,000 g at 4°C. 2 μg biotinylated Fab or IgG were added to 200 μL cell lysate (1–2.5 mg of total protein). 15 μL of beads coupled to an anti-FLAG antibody (M2, Sigma F1804) were added to 200μl lysate as a positive control. The solutions were incubated at 4°C overnight and then 20 μL of pre-equilibrated (by low salt AFC, 10 mM TRIS pH 7.9, 100 mM NaCl, 0.1% NP–40) streptavidin beads (for Fabs, Dynabeads, Life Technologies) and protein A/G-sepharose beads (for IgGs, Dynabeads, Life Technologies) were added. After slow rotation for 2 h at 4°C, streptavidin beads and protein A/G-sepharose beads were washed four times using low salt AFC. Bound proteins were eluted with 100 μL of sample buffer (40% Glycerol, 240 mM Tris/HCl pH 6.8, 8% SDS, 0.04% bromophenol blue, 5% beta-mercaptoethanol) and resolved using gel electrophoresis. Western blots were performed using standard techniques and the detection of tagged antigens detected using the M2 anti-FLAG antibody.

### IP-Mass Spectrometry

Cell lysis and immunoprecipitation was performed as described in IP-WB protocol except the washing steps differed. Antigen-antibody bound streptavidin beads and protein A/G-sepharose beads were washed with high salt AFC buffer 3X and 2X with high salt AFC buffer with no detergent. The immunoprecipitated proteins were eluted with 4x50 μL 0.5 M ammonium hydroxide. Samples were dried and trypsin digestion was performed. Briefly, samples were resuspended in 44 mL of 50 mM of NH_4_HCO_3_ and 1 mL of 100 mM TCEP-HCl (ThermoFisher) was added. After 1 h incubation at 37°C with shaking, the samples were cooled to RT, 1 mL of 500 mM iodoacetamide added and the mixture incubated in the dark at RT for 45 min. 1 mg of trypsin (Promega) was added to each sample and incubated overnight with shaking at 37°C. The digestion was stopped by the addition of 2 mL of acetic acid. The final volume was 50.5 mL. Desalting was performed using ZIP-TIP (Millipore, Catalog# ZTC18M960) according to the manufacturer’s protocol. Samples were dessicated and acidified with 1% formic acid, followed and the peptides were ready to be injected into LC-MS/MS (Orbitrap Velos mass spectrometer; Thermo Fisher Scientific).

### ChIP-Western Blot and ChIP-qPCR

Chromatin immunoprecipitation was performed similarly to previously published reports with some modifications [31, 32]. Briefly, cross-linked HEK293 cells (from 1x15 cm plate) were lysed (1.5 mL) and the DNA fragmented by sonication (Bioruptor; 3*15 cycles, 30s on and 30s off). Cell lysates were clarified by centrifugation for 10 min at 13,000 rpm and the supernatant moved to a new tube. Triton-X was added to final concentration of 0.1% and the lysate was split into 0.2 mL aliquots to which pertinent antibodies were added (2 mg) and the mixtures incubated at 4°C overnight. The next day, appropriate magnetic beads were added as in IP-MS protocol. As one negative control, we added beads alone, and as another, unrelated or failed antibodies. The lysates were rotated for 2 h at 4°C. Beads were washed 5X with RIPA wash buffer (10 mM Tris-HCl ph 8.0, 100 mM NaCl, 1 mM EDTA, 0.5 mM EGTA, 0.1% Na-Deoxycholate, 0.5% N-lauroylsarcosine) and 1X with TE buffer supplemented with 50 mM NaCl. DNA was eluted with elution buffer (50 mM Tris, 10 mM EDTA, 1% SDS) for 20 min at 65°C and moved to a fresh tube. De-crosslinking was carried out at 65°C overnight, followed by RNAse A and Proteinase K digestion. DNA was purified with phenol:chloroform. qPCR was performed using power SYBR green (ABI) on VIIA7 machine (ABI). Primers are listed in the S1 Table.

## Supporting Information

S1 Fig Competitive ELISA. A) Scatter plot of a single point competitive ELISA data obtained for BAZ2B, BRD4, CBX3, CBX5, EP300, JMJD2A, JMJD2C, JMJD3, L3MBTL2, PHF8, PRDM4, SFMBT2, SMARCA4 where 96 individual Fab-phage clones for each of the targets were tested in high-throughput format (gray circles). On the Y-axis OD 450 nm is plotted for direct binding of the Fab-phage to biotinylated antigen immobilized on a neutravidin-coated ELISA plate and probed with an anti-M13 phage antibody. In competitive binding experiment Fab-phage is preincubated with 20 nM soluble antigen and then allowed to bind to the antigen coated plate for 15 min, washed and probed with an anti-M13 phage antibody similarly to direct binding assay (see methods section). On X-axis competition ratio is shown and calculated as OD 450 nm of competitive binding divided by the OD 450 nm of direct binding signal. Fab clones in top left quadrant are considered passing when OD 450 nm signal is higher then 0.1 AU and competition ratio is lower then 0.7 (red dashed cutoff lines). Clones in bottom right quadrant are considered as failing. This assay provides an estimate of both affinities in Fab-phage format and expression levels. Red circles denote clones picked for SPR binding analysis in protein format. (B) Distribution of dissociation constants (K_D_) measured by surface plasmon resonance for the clones picked used single point competitive assay. Most Fabs have dissociation constants in single digit nanomolar region while 15% of binders fall into sub-nanomolar range, only 5% exceed 20 nM set threshold.
(TIF)

S2 Fig Heat denaturation step. SDS-PAGE analysis of 4 Fabs purified on protein A-sepharose (left). A heat denaturation step was included to remove the smaller fragments and SDS-PAGE analysis of heat-treated Fabs is shown on the right. Most of the smaller Fab fragments have been cleared by the heat treatment.
(TIF)

S3 Fig Chromatin immunoprecipitation. (A) Overview of ChIP-WB and ChIP-qPCR protocol. (B) ChIP-WB results for Fabs and IgGs against 5 different targets. Epitope-tagged cell lysates (indicated on the left) were used for immunoprecipitation according to the ChIP protocol. Following immunoprecipitation, protein:DNA complexes were eluted with SDS protein sample buffer and Western blot was performed. The immunoprecipitated proteins were detected with anti-M2 antibody against epitope tag. (C) ChIP-qPCR results for four targets (n = 2; error bars indicate biological replicas). Immunoprecipitated DNA was amplified with primers against the genomic loci previously known to be occupied by the target genes. These same primers were used to amplify immunoprecipitated DNA from unrelated Fabs as control. BRD1 is enriched at the promoter and transcription start site (TSS) of GATA1 and TAL1 but L3MBTl2 and SFMBT2 do not show enrichment at these loci (i). SFMBT2 is enriched at the promoter of HOXB13 and HOXC13 while L3MBTL2 is enriched at HOXC13 and RPA2 promoters (ii). CBX1 but not CBX2 is enriched at the alpha satellite sequences (iii). BRD4 is enriched at the promoters of CCND1 and CDK7 (iv).
(TIF)

S1 Table List of primers used for ChIP-qPCR. (DOCX)
