# Supplementary figures and images for: Optimizing Production of Antigens and Fabs in the Context of Generating Recombinant Antibodies to Human Proteins
Source: PLoS One. 2015 Oct 5;10(10):e0139695. doi: 10.1371/journal.pone.0139695 (PMC4593582; doi:10.1371/journal.pone.0139695)

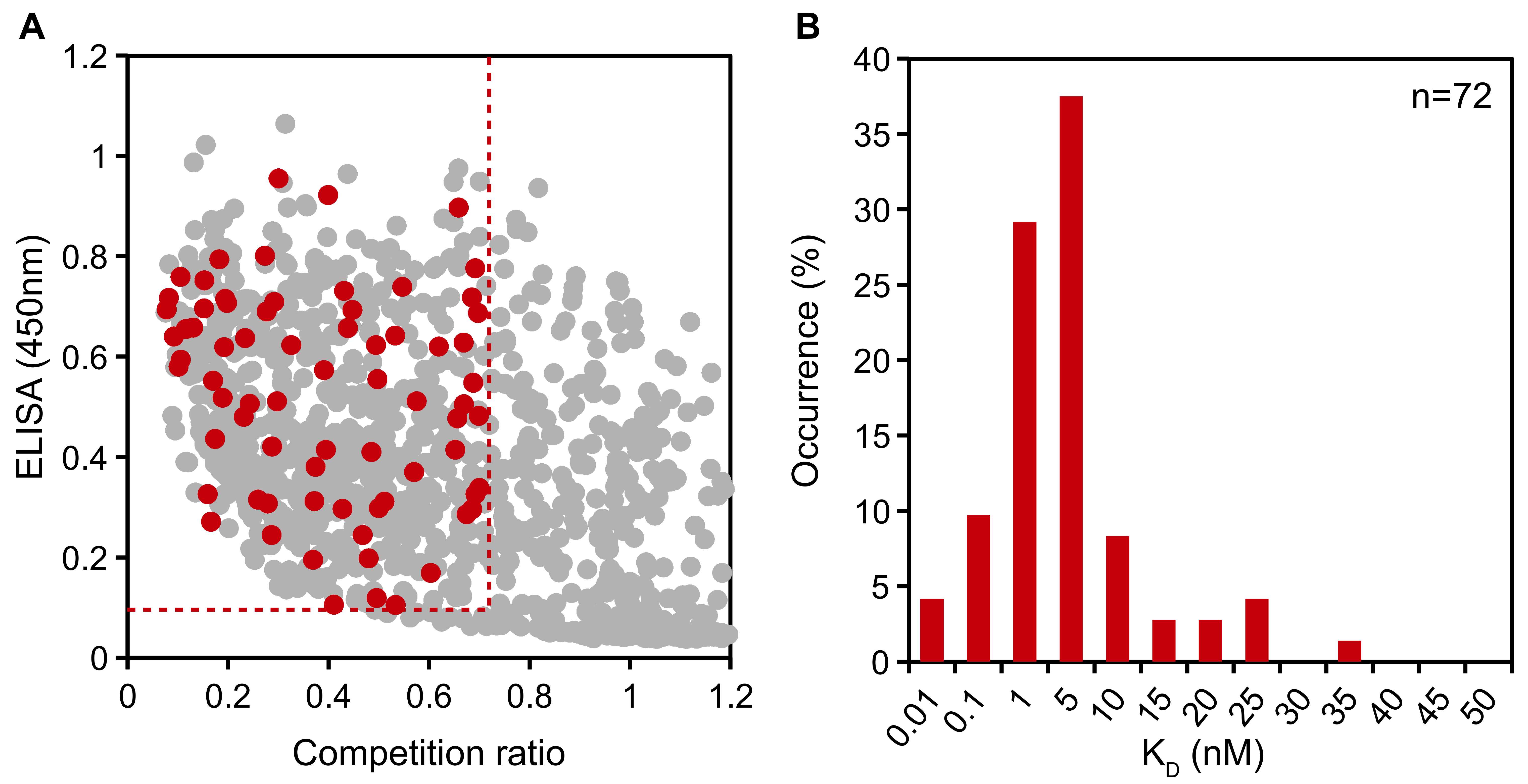

Supplement: S1 Fig — A) Scatter plot of a single point competitive ELISA data obtained for BAZ2B, BRD4, CBX3, CBX5, EP300, JMJD2A, JMJD2C, JMJD3, L3MBTL2, PHF8, PRDM4, SFMBT2, SMARCA4 where 96 individual Fab-phage clones for each of the targets were tested in high-throughput format (gray circles). On the Y-axis OD 450 nm is plotted for direct binding of the Fab-phage to biotinylated antigen immobilized on a neutravidin-coated ELISA plate and probed with an anti-M13 phage antibody. In competitive binding experiment Fab-phage is preincubated with 20 nM soluble antigen and then allowed to bind to the antigen coated plate for 15 min, washed and probed with an anti-M13 phage antibody similarly to direct binding assay (see methods section). On X-axis competition ratio is shown and calculated as OD 450 nm of competitive binding divided by the OD 450 nm of direct binding signal. Fab clones in top left quadrant are considered passing when OD 450 nm signal is higher then 0.1 AU and competition ratio is lower then 0.7 (red dashed cutoff lines). Clones in bottom right quadrant are considered as failing. This assay provides an estimate of both affinities in Fab-phage format and expression levels. Red circles denote clones picked for SPR binding analysis in protein format. (B) Distribution of dissociation constants (KD) measured by surface plasmon resonance for the clones picked used single point competitive assay. Most Fabs have dissociation constants in single digit nanomolar region while 15% of binders fall into sub-nanomolar range, only 5% exceed 20 nM set threshold. (TIF) [file pone.0139695.s001.tif]

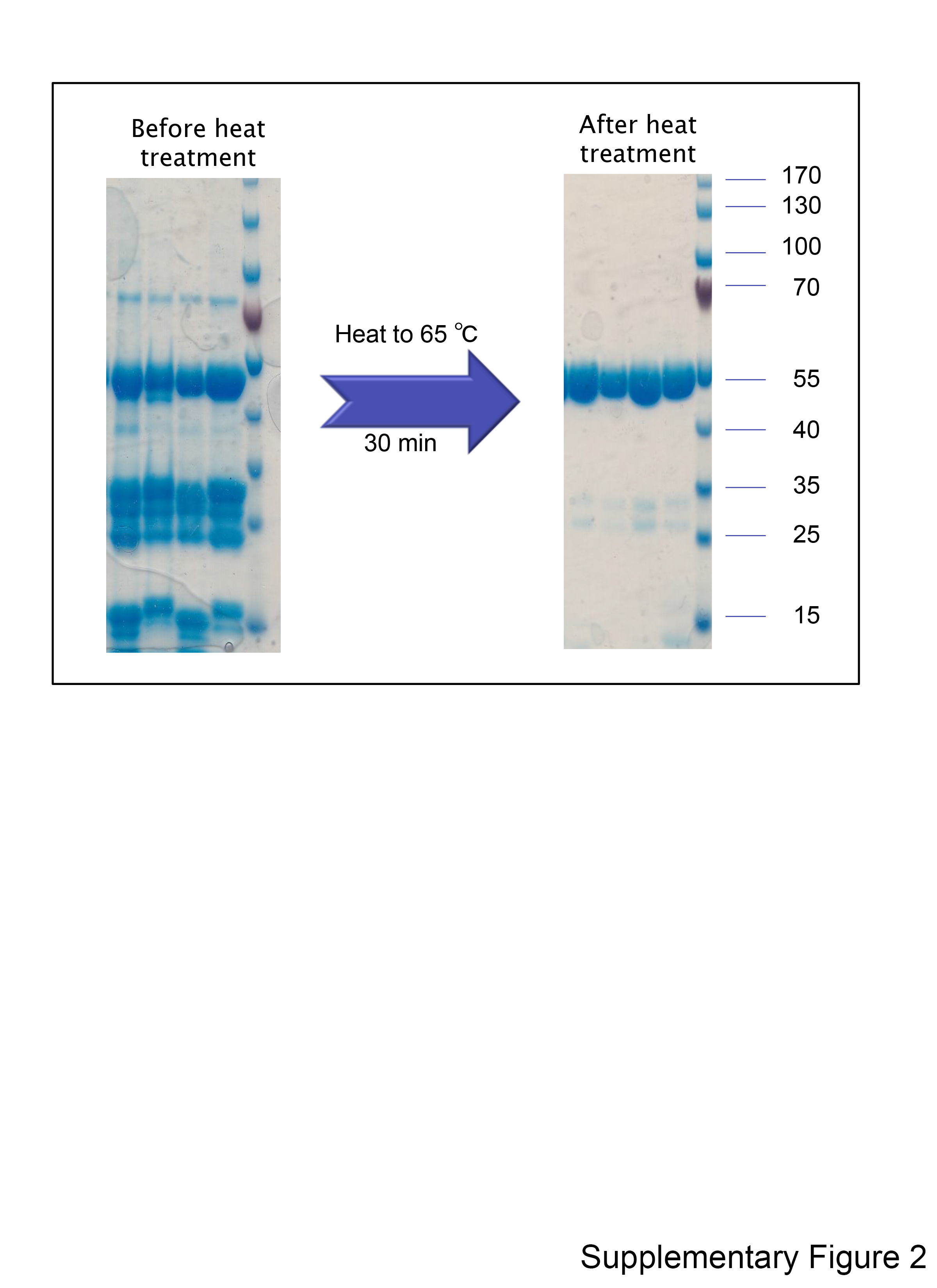

Supplement: S2 Fig — SDS-PAGE analysis of 4 Fabs purified on protein A-sepharose (left). A heat denaturation step was included to remove the smaller fragments and SDS-PAGE analysis of heat-treated Fabs is shown on the right. Most of the smaller Fab fragments have been cleared by the heat treatment. (TIF) [file pone.0139695.s002.tif]

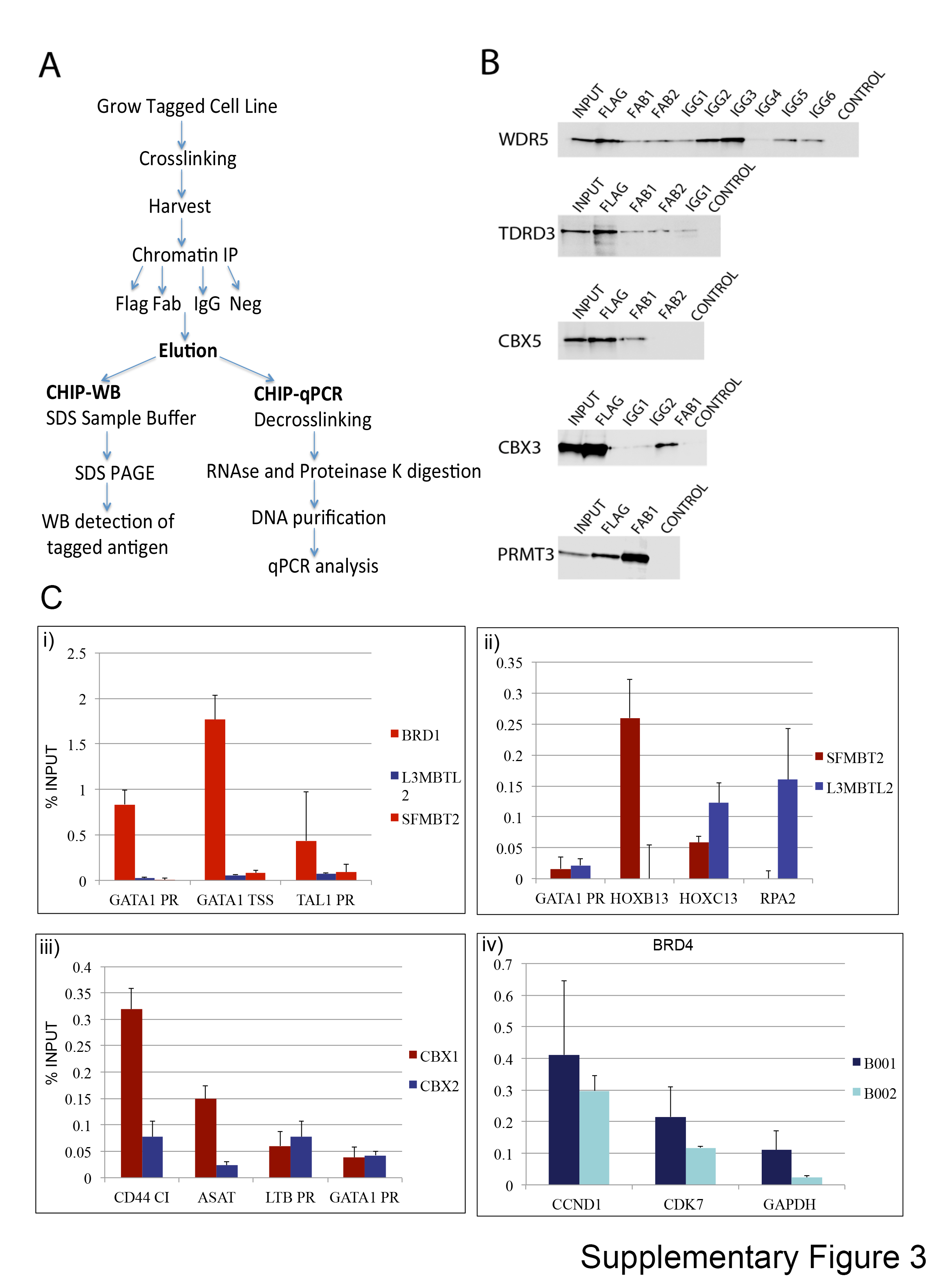

Supplement: S3 Fig — (A) Overview of ChIP-WB and ChIP-qPCR protocol. (B) ChIP-WB results for Fabs and IgGs against 5 different targets. Epitope-tagged cell lysates (indicated on the left) were used for immunoprecipitation according to the ChIP protocol. Following immunoprecipitation, protein:DNA complexes were eluted with SDS protein sample buffer and Western blot was performed. The immunoprecipitated proteins were detected with anti-M2 antibody against epitope tag. (C) ChIP-qPCR results for four targets (n = 2; error bars indicate biological replicas). Immunoprecipitated DNA was amplified with primers against the genomic loci previously known to be occupied by the target genes. These same primers were used to amplify immunoprecipitated DNA from unrelated Fabs as control. BRD1 is enriched at the promoter and transcription start site (TSS) of GATA1 and TAL1 but L3MBTl2 and SFMBT2 do not show enrichment at these loci (i). SFMBT2 is enriched at the promoter of HOXB13 and HOXC13 while L3MBTL2 is enriched at HOXC13 and RPA2 promoters (ii). CBX1 but not CBX2 is enriched at the alpha satellite sequences (iii). BRD4 is enriched at the promoters of CCND1 and CDK7 (iv). (TIF) [file pone.0139695.s003.tif]
