# Supplementary material for: Optimizing Production of Antigens and Fabs in the Context of Generating Recombinant Antibodies to Human Proteins
Source: PLoS One. 2015 Oct 5;10(10):e0139695. doi: 10.1371/journal.pone.0139695 (PMC4593582; doi:10.1371/journal.pone.0139695)
Supplement: S1 Table — (DOCX) [file pone.0139695.s004.docx]

Supplementary Table 1. **Primers used for qPCR**

| **Name** | **Primer** | **Reference** |
| --- | --- | --- |
| GATA1 PROM F | GCTTAGCCTGGGTCATCAAG | Mishima et al., 2011 |
| GATA1 PROM R | GGATGTGGCTGTACCCATTT |  |
| TAL1 PROM F | CTTTCCCCCTTTGTTGGTCT | Mishima et al., 2011 |
| TAL1 PROM R | AGGGGGCTTGGAGAGAGATA |  |
| GATA1 TSS F | CTTGTCTTTGCCCCACTCTC | Mishima et al., 2011 |
| GATA1 TSS R | TACTGAGCAGGCAGGGAGTT |  |
| GAPDH PROM F | TACTAGCGGTTTTACGGGCG |  |
| GAPDH PROM R | TCGAACAGGAGGAGCAGAGAGCGA |  |
| CCND1.PR1.F | CTGAGATTCTTTGGCCGTCT |  |
| CCND1.PR1.R | GCTCTGCAGTAGGGGACAAC |  |
| CDK7.PR1.F | ACCTACCAAACCCCGAACAT |  |
| CDK7.PR1.R | TCTTCAATGGCTGCTGTGTC |  |
| CD44_C1F | CTCCGGACACCATGGACAAGTTT | Saint-Andre et al., 2011 |
| CD44_C1R | CCAATGCACTTCGCAGACAGCTCA |  |
| LTB PROM F | TCCATCCCTCAAGATTCCAG |  |
| LTB PROM R | AGGTTTCCAATGTGGTTTGC |  |
| A-SAT.F | TCATTCCCACAAACTGCGTTG | Gopalakrishnan et al., 2009 |
| A-SAT.R | TCCAACGAAGGCCACAAGA |  |
